# Supplementary figures and images for: Cells tile a flat plane by controlling geometries during morphogenesis of Pyropia thalli
Source: PeerJ. 2017 May 11;5:e3314. doi: 10.7717/peerj.3314 (PMC5429738; doi:10.7717/peerj.3314)

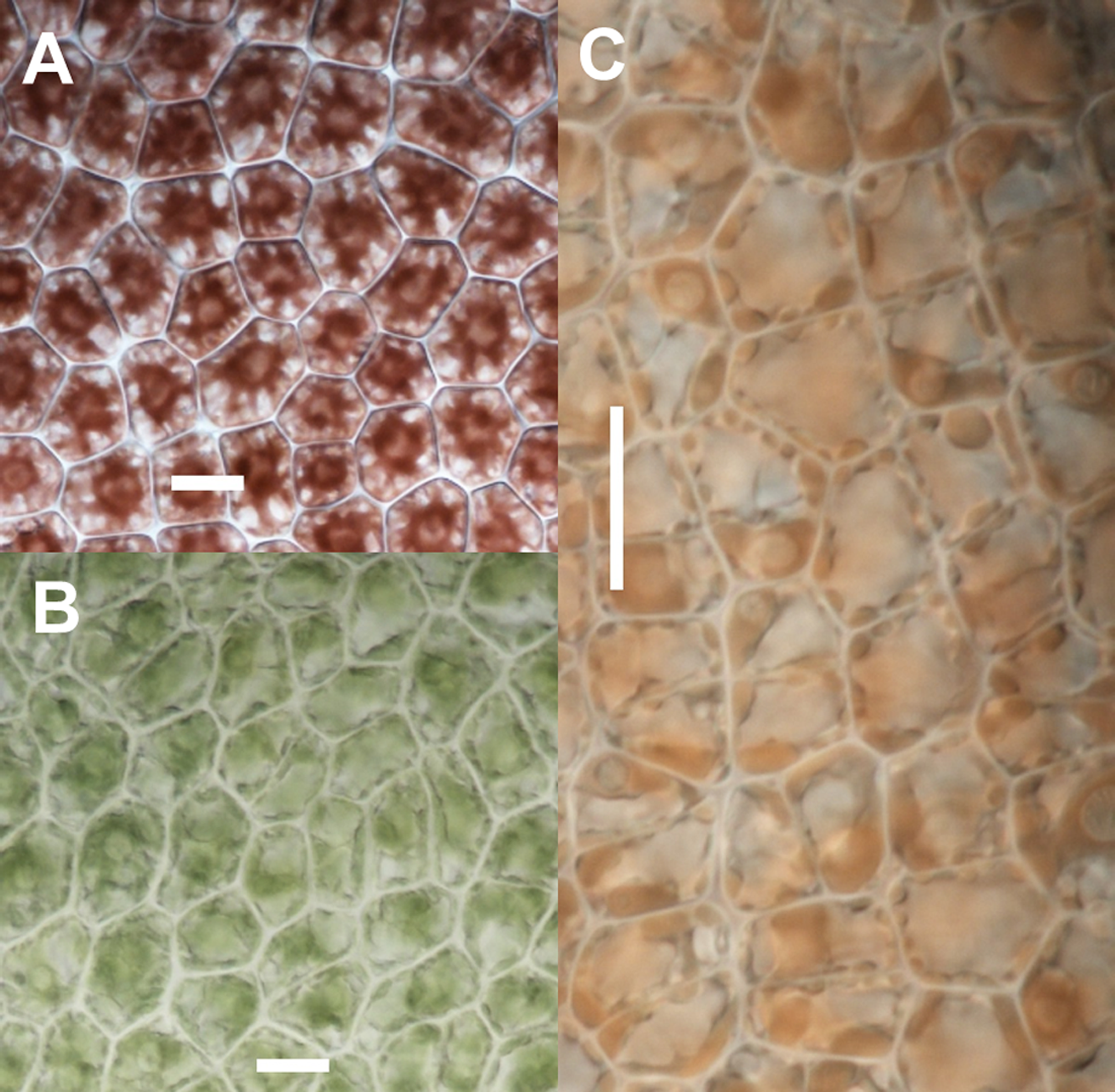

Supplement: Figure S1 — (A) Z-61 in red-brown, (B) G-2 in green, and (C) O-9 in orange. All scale bars are 20 µm. [file peerj-05-3314-s001.png]
